# Promoting Inclusion and Mitigating Digital Divide Impacts in International Development Research Teams: The PIRL Project

**DOI:** 10.1057/s41287-026-00754-8

**Published:** 2026-06-15

**Authors:** Lynn Cockburn, Ellen Murray, Julius Tanyu Nganji, Pedro H. T. Q. de Almeida, Soomin Lee, Che Dieudonne Nkemchap, Louis Mbibeh, Kometa Sunday Shende, Nde Vitalis Tangeh, Marc Stephan Nkouly

**Affiliations:** 1 University of Toronto, Toronto, Canada; 2 McMaster University, Hamilton, Canada; 3 Ellen Murray Consulting, Toronto, Canada; 4 University of British Columbia, Vancouver, Canada; 5 Coordinating Unit of Associations of Persons with Disabilities, Bamenda, Cameroon; 6 University of Bamenda, Bamenda, Cameroon; 7 STARC, Bamenda, Cameroon

**Keywords:** Digital divide, Research teams, Capabilities approach, Inclusive international teams, Disability studies, ICT4D

## Abstract

To conduct high-quality international development research, teams must fully include diverse researchers who contribute to knowledge creation. This paper highlights the often-overlooked digital divide within international research teams and examines how systematically creating a collaborative plan and process that addresses use of Information and Communication Technologies (ICTs) within research teams can improve inclusion, particularly for researchers with disabilities, from different geographic locations and income levels. We used a cross-sectional, mixed methods, participatory research approach to collect data from members of the PIRL Project, who lived primarily in North America and Sub-Saharan Africa. Members included community and academic researchers, some with disabilities, low incomes, and differing educational and employment backgrounds, all with a shared objective to collaboratively improve research capabilities. Mitigation strategies to overcome digital divide inequities included collaborative ICT planning, use of smartphones, free apps, and training. The Capability Approach framed reflexive analysis of the results.

## Résumé

Pour mener des recherches en développement international de haute qualité, il est essentiel d’inclure pleinement des chercheurs issus de divers horizons, qui contribuent à la création de connaissances. Cet article met en lumière la fracture numérique, souvent négligée, au sein des équipes de recherche internationales et examine comment l’élaboration systématique d’un plan et d’un processus collaboratifs, prenant en compte l’utilisation des technologies de l’information et de la communication (TIC) au sein des équipes de recherche, peut favoriser l’inclusion, en particulier pour les chercheurs en situation de handicap, issus de différentes régions géographiques et de niveaux de revenus variés. Nous avons adopté une approche transversale, mixte et participative pour recueillir des données auprès des membres du projet PIRL, résidant principalement en Amérique du Nord et en Afrique subsaharienne. Les membres comprenaient des chercheurs communautaires et universitaires, certains en situation de handicap, à faibles revenus, et issus de parcours éducatifs et professionnels divers, tous animés par l’objectif commun d’améliorer collectivement les capacités de recherche. Les stratégies d’atténuation visant à surmonter les inégalités liées à la fracture numérique comprenaient la planification collaborative des TIC, l’utilisation de smartphones, d’applications gratuites et la formation. L’Approche par les Capabilités a servi de cadre à l’analyse réflexive des résultats.

## Resumen

Para llevar a cabo investigaciones de desarrollo internacional de alta calidad, los equipos deben incluir plenamente a investigadores diversos que contribuyan a la creación de conocimiento. Este artículo destaca la brecha digital, a menudo pasada por alto, dentro de los equipos de investigación internacionales y examina cómo la elaboración sistemática de un plan y un proceso colaborativos que aborden el uso de Tecnologías de la Información y la Comunicación (TIC) en los equipos de investigación puede mejorar la inclusión, especialmente para investigadores con discapacidades, de diferentes ubicaciones geográficas y niveles de ingresos. Se utilizó un enfoque de investigación participativa, transversal y de métodos mixtos para recopilar datos de los miembros del Proyecto PIRL, quienes residían principalmente en América del Norte y África Subsahariana. Los miembros incluían investigadores comunitarios y académicos, algunos con discapacidades, bajos ingresos y diferentes trayectorias educativas y laborales, todos con el objetivo común de mejorar colaborativamente las capacidades de investigación. Las estrategias de mitigación para superar las desigualdades derivadas de la brecha digital incluyeron la planificación colaborativa de las TIC, el uso de teléfonos inteligentes, aplicaciones gratuitas y capacitación. El Enfoque de Capacidades sirvió como marco para el análisis reflexivo de los resultados.

To conduct high-quality collaborative international development research, teams must include diverse researchers (Apgar et al. 2023; Hughes et al. 2020). Inclusivity requires intentional design and implementation strategies to increase the agency and participation of researchers from historically marginalized groups like those with disabilities (Hughes et al. 2020). Equitable access to ICT by international researchers is often assumed and needs questioning, yet there appear to be few studies that explore the real-world disparities of digital inequalities in research processes. For example, even in well-resourced settings, there are often unacknowledged yet distinct digital differences across individuals, communities, and institutional and geographic locations (Fransman et al. 2021). Evidence about access to information and communication technology (ICT) resources and the digital divide within international development research teams remains nascent. Reflecting the awareness that technology in itself is insufficient for creating new knowledge and significant social change, researchers such as Álvarez-Camacho et al. (2021), Ganapathy et al. (2024), Masiero (2022), and Wolbring and Lillywhite (2021) have asked how researchers can shape more fair, inclusive, and equitable research-related structures and processes. We want to turn these questions inward, within research teams, understanding that they operate nested in structures and systems, much like Palladino et al. (2023) describe in their reflections on international, interdisciplinary, and cross-sector collaborations.

In this paper we focus on understanding the role of ICTs as an indicator of equity within teams and asked ourselves what more inclusive and equitable processes could be used to attend to individuals’ experiences, to support interpersonal dynamics within a research team, and to create an inclusive, participatory research team approach. We echo the challenges articulated by Ganapathy et al. (2024) and Heeks (2022) for development researchers to examine social, institutional, and political structures within their own teams.

We report on a study to understand ICT use and capability sets in a complex international development research community, as a partial response to concerns about inclusion and access to ICT and participation in research. The study examines research collaboration between international researchers, including the intersectional experiences of those living with disabilities, with low-incomes, and in low and middle-income countries (LMIC). The paper reflexively examines effects of structural inequities and the digital divide on participation and collaboration in research. The feasibility of intentionally developing and using an ICT Project Plan to mitigate digital divide effects on international teams is suggested as an effective strategy.

To provide context, here we briefly describe the focus of this paper, then provide more detail in the Methods section. The larger study, and this paper, were completed by the Partnerships for Inclusive Research and Learning (PIRL) Project. PIRL was formed by several Cameroonian and Canadian researchers who had collaborated on previous projects addressing a range of structural, community, and individual issues (Burris et al. 2021; Cockburn et al 2019; Mbibeh et al. 2020; Nganji and Cockburn 2019); as often happens with research teams, some researchers identified areas of research for future study while completing those previous studies. The intention with this project was to form a diverse team that could be intentional and international in examining connections between in person and virtual collaborations that are embedded in complex research systems and structures (Ganapathy et al. 2026; Palladino et al. 2023), including addressing equity and access to ICT. PIRL included academic researchers, community researchers from non-governmental organizations, researcher-activists representing disability advocacy organizations, and researchers who worked for private companies providing ICT or accessibility services. The partnership began with 18 co-applicants and collaborators, primarily from 5 partner organizations, who created a community of practice; several others, including undergraduate and graduate students, joined over the four years of the project (2019–2023). The use of “we/our” in this paper refers to the core team of about 25 PIRL researchers who were active in PIRL sub-projects over the 4 years of the project. The PIRL Network encompassed the 100-plus PIRL members involved in PIRL activities (https://icdr.utoronto.ca/our-community/cameroon).

## Background

Consensus in international development recognizes that locally based researchers are crucial to international projects (Kaufman et al. 2022; Salm et al. 2025) but often fails to recognize that community and academic researchers in LMIC face challenges related to ICT access and use (Álvarez-Camacho et al. 2021; Ganapathy et al. 2026). Collaboration can be further complicated when researchers, especially those from high income countries or who are not familiar with access and inclusion efforts, bring misconceptions, expectations, and “deficit” biases to projects (Africa Evidence Network 2021; Fransman and Newman 2019). One result is the exclusion of researchers with disabilities, both in academic life (Mellifont et al. 2019; University of British Columbia 2021) and related to research on development projects (Stein and Lord 2021; UNDESA 2017). The same deficit biases apply to researchers with low incomes and who face barriers to ICT access and collaboration (Africa Evidence Network 2021; Álvarez-Camacho et al. 2021; Palladino et al. 2023).

Development literature uses many terms to describe disparities in ICTs, services, income, and opportunities, including “developing country”, “Global South”, “low- or-middle income country (LMIC)” or “developed country”, “Global North”, and “high-income country (HIC)”. Here we use the terms LMIC and HIC, although these terms centre economic factors, obscuring and diminishing the broad, complex effects of colonialism and globalization on social participation and collaboration (Africa Evidence Network 2021; Haug et al. 2021; Khan et al. 2022; Lencucha and Neupane 2022; Palladino et al. 2023).

The United Nations (UN) describes development as a multidimensional undertaking focused on social and economic progress, which improves people’s standard of living, empowers marginalized groups, and protects environment and democratic processes (UN Library 2023). This perspective is grounded in economist Amartya Sen’s Capability Approach (CA) where development must increase freedoms (Pazzanese 2021; Sen 1999). Capabilities are the range of choices an individual can realistically act on; when an available opportunity is selected, this realized capability is called a functioning (Sen 1999). The CA posits development as a process to increase individuals’ capabilities and freedoms to be and do what they chose, especially for those with smaller capability sets (Oosterlaken 2015).

The CA questions if individual lives (not solely economic indicators) are improved by development projects (Sen 1999). For example, does a project increase capabilities or freedoms for being healthy, being literate, voting, or working (Oosterlaken 2015; Sen 1999)? The CA proposes that capabilities can be increased by changing the *input resources* available, such as income, clean water, and ICT tools. Capabilities can also increase by changing the individual’s *conversion factors* which influence how an available input resource becomes a capability (Oosterlaken 2015; Sen 2004; Garcés Velástegui 2020). The three categories of conversion factors are environmental (e.g., physical or built surroundings), personal (e.g. internal to a person, impairments), and social (e.g., public policies, social norms) (Robeyns 2005).

## Barriers for Accessing ICTs: The Digital Divide

The term digital divide describes inequitable access to information technology and reflects the concept of intersectionality, an evolving understanding that the impacts of social identities and locations must be examined together, not separately (Carbado et al. 2013). The concept of a “digital divide” is changing to be more inclusive of concepts such as digital justice and adverse digital incorporation (Heeks 2022; Masiero 2022). Numerous reports show the digital divide is experienced differently based on the intersections of gender, health, disability status, age, education, urban or rural location, and other social identities (Faith and Hernandez 2024; Fisk et al. 2022; GSMA 2022, 2025a, b; Heeks 2022; International Telecommunication Union of the UN (ITU) 2021a, b, c). Examples of the digital divide include social norms that exclude women from access to ICT; decreased access to ICT infrastructure and resources in LMIC and rural areas; national policies that limit internet access; and researchers with disabilities excluded by teammates who lack knowledge about inclusion (GSMA 2022; International Telecommunication Union of the UN (ITU) 2021a, b, c; Mellifont et al. 2019; Wolbring and Lillywhite 2021). Faith and Hernandez’s work (2024) is a reminder that the digital divide affects many people in HICs, describing a mobile underclass who face barriers obtaining employment and government benefit supports when they only have access to a smartphone or tablet with limited capabilities or they lack digital skills.

Access to ICTs impacts researcher capability sets. For example, requisite researcher tools include apps for online meetings, communication, cloud-based document management and presentations, sharing on social media, data collection and analysis, gathering information, bibliographic apps, and the skills, time, and infrastructure to use them. The PIRL ICT Plan examined ways in which researchers could consider their ICT resources and work collaboratively to help mitigate digital divide challenges to increase capabilities and reach desired outcomes (Fang et al. 2019; Kleine 2011).

## Methods

PIRL was a participatory research project (Hall and Tandon 2017) which used a mixed methods design. The roles involved in PIRL are shown in Table 1.

**Table 1:** Overview of people involved in the PIRL Project

People	Number	Description of group	Data created
PIRL ***Core Team***	25 (approx.)	Decision making and responsibility for the overall participatory research project, including formulating and implementing the PIRL ICT Plan, and PIRL subprojects such as the ICT Research Study	Project documentation, records of decisions made, and field notes
PIRL Project ***Members*** (PIRL Network)	100 + (varied over the 5 years of the project)	Shared information about ICTs and other research topics related to the goals of PIRL Project	Meeting minutes and recordings, field notes, and research article summaries
PIRL ICT Research Study ***Participants*** Survey ***Respondents*** ***Interviewees***	78 (survey and interviews) 60 (survey) 18 (interviews)	PIRL members who volunteered to participate in the ICT Research study, a PIRL subproject	Quantitative and qualitative data from survey Interview transcripts
PIRL ICT ***Writing Group***	10	Members of PIRL project who engaged in reflexive writing to prepare this paper	Meeting minutes, article summaries, data analysis, drafts and final manuscript

The research questions were “When, how, and why are information and communication technologies (ICTs) used in international research teams focused on inclusive development?” and “How can development research become more inclusive, despite structural inequities and the digital divide?”. We use three PIRL subprojects to answer these questions (Table 2): formulating and analysing the PIRL ICT Plan; an embedded, formal research project which collected data from PIRL network members about ICT access and use; and the work of this paper’s PIRL ICT writing group.

**Table 2:** PIRL subprojects discussed in this paper

Subproject	Purpose	People involved
PIRL ICT Plan	(1) To develop a detailed plan of ways the PIRL Project could use ICT and collaborate to help mitigate digital divide challenges (2) To collectively decide on ICT use to enable inclusion and access for all PIRL members	Core PIRL Team
PIRL ICT Research Study	(1) To explore the use of ICTs related to team collaboration, using an internet-based survey and interviews (2) To provide a shared, participatory research experience for PIRL Project members	Core PIRL Team Survey Respondents Interviewees
PIRL ICT Writing Group	To collaboratively write this paper about ICT use in research teams, using the capability approach (CA)	PIRL ICT Writing Group Core PIRL Team

### The PIRL ICT Plan

One of the first PIRL priorities was to develop an ICT Plan for promoting strategies for inclusion. Decisions focused on applications that fostered inclusion and optimized collaboration, rather than being driven by ICT availability of or for privileged team members. The plan was iteratively reviewed and is further explored below.

### The ICT Research Study: Survey and Interviews

In addition to gathering useful information, the survey and interview subprojects were designed to provide research and community building opportunities among the team. An internet-based survey (Menon and Muraleedharan 2020) to explore the use of ICTs for research team collaboration was developed through an inclusive, participatory approach. Given that we desired an accessible survey and that some respondents would use screen readers, we compared potential survey hosting platforms based on accessibility criteria (David Berman Communications 2011). We choose SurveyMonkey, a widely used survey platform with good accessibility features, now meeting WCAG2 (2025) standards for web-based content (“Accessibility When Using SurveyMonkey”), https://help.surveymonkey.com/en/surveymonkey/create/accessibility/).

Questions were adapted from the Canadian Internet Use Survey (“Canadian Internet Use Survey 2018,” https://www.statcan.gc.ca/en/statistical-programs/instrument/4432_Q2_V2) and team members’ international experiences. The survey included multiple choice and open-ended questions covering demographics, occupations, technology ownership, ICT access, social media use, barriers to technology, and internet usage. Single choice answers included geographical location, gender, and income ranges. Disability-related information was gathered through three methods: the Washington Group short set of questions on functioning (covering walking, hearing, seeing, memory, self-care, and communication) (The Washington Group on Disability Statistics 2024); self-identification as living with a disability, and descriptive comments on impairments and disability. Survey invitations were emailed three times to more than 100 PIRL Network members over one month beginning September 4, 2020.

The semi-structured interview guide of approximately 20 questions was participatively generated from survey responses. Three questions specifically focused on ICT use. The Washington Group Short Set questions were included (The Washington Group on Disability Statistics 2024). As an international project we had planned for both virtual and face-to-face research interviews. However, all interviews were converted to online format using Zoom due to restrictions imposed by the COVID-19 pandemic. Although we took steps to support those who might have difficulty with Zoom, not offering an in-person option might have shaped whose voices were included in this data or their responses. Multiple invitations to be an interviewer or interviewee were sent to PIRL Network members between October 2020 and July 2021. Interviewers were involved in a training process developed by the team which included many hours of discussions related to putting theory into practice and conducting mock research interviews.

For interviewee selection, a stratified purposive sampling technique (Palinkas et al. 2015) was used to ensure that marginalized groups based on geographic location, gender, and disability were represented. We aimed to have approximately 10 from each of the identified marginalized groups (living in LMIC, women, living with disability); using an intersectional approach meant that one person could be in one, two, or three of the groups (e.g., A woman living with disability in a LMIC). All interviewees provided consent.

### Approvals

The PIRL Steering Committee approved the survey and the interview guide. Ethical approval was obtained from the University of Bamenda (Cameroon) and the University of Toronto (Canada). The survey and interview guide are available from the authors.

### Analysis and Writing

Data analysis and writing sub-project teams provided PIRL members opportunities to engage in the participatory research process. These groups shared literature, analyzed data, and collaborated on theme development. The PIRL writing team for this paper held Zoom meetings biweekly for approximately one year. Meetings had clear guidelines (e.g., for data analysis and writing contributions) and topics, and used WhatsApp, emails, Google Drive folders and Docs, and a Zotero bibliography.

Survey data was downloaded into Microsoft Excel then deduplicated, cleaned, anonymized, and coded. Statistical analysis was performed using SPSS (a data analysis program) to generate frequency distributions and Chi-squared tests of independence to look for significant relationships between location, gender, disability, or income, and the uses of ICTs. Descriptive analysis of the survey results was done using SPSS, Google Sheets, Microsoft Word, and Excel. Survey disability codes were consolidated from survey responses about living with a disability and responses to the Washington Group questions (The Washington Group 2021).

The data analysis and writing team members transcribed and coded interviews (Flicker and Nixon 2015). Each interview transcript was coded independently by at least two people and then discussed with other team members. The coding results were entered into NVivo, allowing for thematic analysis. Both the SPSS and NVivo results were exported into Word and Google docs to share with team researchers/writers who had limited software access.

Finally, to write this paper our team aggregated the survey results, the interview themes, and documents related to the development and implementation of the PIRL ICT Plan. Throughout this process we used the Capability Approach, seeing ICTs as research resource inputs and looking for relevant conversion factors (Haenssgen and Ariana 2018; Oosterlaken 2015; Robeyns 2005; Sen 1999).

## Findings

The findings are presented in three sections. First the demographic information about the survey respondents and interviewees is provided, then two themes are described. The first theme is “Paying attention to the nuanced complexities of the Digital Divide: ICT access affects researchers”, and the second is “Taking Action: Striving to Increase the Capability Sets of Researchers”.

### Demographics

Table 3 shows the demographic details for the PIRL survey respondents and the 18 interviewees. Seventy people attempted the 72-question survey, with 60 valid responses remaining after removal of incomplete responses. Questions about employment, location, education, income, and disability status were completed by 46 to 48 respondents.

**Table 3:** Study participants

		Africa (n)	North America and Europe (n)	Total (n)
SURVEY RESPONDENTS WHO PROVIDED DEMOGRAPHIC INFORMATION Survey frequency results varied by question number				
**Location** (n = 48)		33	15	48
**Gender** (n = 48)	Female	15	8	23
	Male	18	7	25
	Other	0	0	0
**Education level** (n = 46)	Secondary	2	1	3
	Bachelor’s	12	2	14
	Master’s/professional degree	9	5	14
	PhD	8	7	15
**Disability** (n = 47)	Female with disability	4	3	7
	Female without disability	11	5	16
	Male with disability	4	1	5
	Male without disability	13	6	19
**Income level** (n = 42)	Low	12	5	17
	Medium	4	0	4
	High	12	9	21

INTERVIEWS (n = 18)				
**Location**		10	8	18
**Gender**	Female	5	5	10
	Male	5	3	8
**Education level**	Secondary	1	0	1
	Bachelor’s	4	2	6
	Master’s/professional degree	2	4	6
	PhD	3	2	5
**Disability**	Female with disability	2	2	4
	Female without disability	3	3	6
	Male with disability	1	3	4
	Male without disability	2	2	4
**Income level**	Not asked in interviews	–	–	–

Survey respondents were engaged in a range of occupations such as academics, community researchers, students, job seekers, disability activists, physiotherapists, occupational therapists, and ICT professionals. To reflect the absolute differences in income reported, category codes for low, medium, or high personal income were developed separately for LMIC and HIC currencies. The demographic results showed many PIRL researchers had some privilege regardless of whether they lived in a LMIC or a HIC.

Using the Washington Group broad categorization, where disability is the result of difficulty in functioning (The Washington Group 2021), 12/47 (26%) survey respondents were coded as living with a disability. Seven of the 18 interviewed PIRL researchers lived with disabilities: four had vision impairments, two had mobility impairments, one had an unspecified invisible disability. The number of survey respondents with disabilities, at 26%, was more reflective of disability in the general population, estimated to be between 15 to 25% (World Health Organization 2022), than in many research groups, although data about disability in research groups can be difficult to find (Johnston and Sanscartier 2019). Estimates are that between 2 and 10% of university researchers live with a disability (Brown and Leigh 2018; Mellifont et al. 2019; University of Toronto 2021).

### Paying Attention to the Nuanced Complexities of the Digital Divide: ICT Access Affects Researchers

This theme acknowledges that the digital divide affects international research team members differently, depending on the technology inputs available and personal conversion factors, which affected inclusion and collaboration in a range of nuanced ways, limiting or expanding PIRL members’ potential in research. All PIRL research participants had access to smartphones and many also had a personal computer with internet access.

All respondents used phone calls and email. Networking with WhatsApp and social media was nearly ubiquitous. Some researchers reported their ICT access was insufficient to permit them to do the research work they wanted to do, due to input resource limitations (e.g. low incomes, limited ICT device capacity, minimal software access, cell phone service challenges, lack of screen readers), or to personal conversion factors (e.g. lack of technical knowledge, living with an impairment that was not accommodated). Some sighted PIRL members did not know what screen readers were or how others used them, and therefore could not ensure they produced accessible materials. About a quarter of the PIRL team identified as researchers with disabilities; their experiences are discussed in relation to other identities below.

We analysed individual access to ICT devices in the survey responses. All 60 (100%) survey respondents had a smartphone with internet access. However, 9/60 (15%) had no personal computer, so their internet-enabled smartphone was the only personal device used for research activities. When asked about the types of computers they owned, 50/60 (83%) respondents indicated a laptop, with 12/60 (20%) owning both a desktop computer and laptop, and 13/60 (22%) a tablet. From the possible responses of laptop, desktop, or tablet, 19/60 (32%) owned two or three of these devices. For those who did not have a computer, comments emphasized financial constraints. For example, one survey respondent wrote “access to financial cash is very limited [for] women with disabilities”. Of the 9 respondents without a computer, 6 lived in a LMIC and 3 did not state their location.

Ownership of a computer did not ensure that the device was functionally useful as a research tool, and several respondents, especially from African countries, identified challenges. One survey respondent wrote: “[my computer] is very slow [compared to colleagues in HIC]—the storage capacity is not enough, battery is not strong, and it doesn’t last long”. Participants recognized the importance of having a computer to be able to engage in research work. Interviewee P12 stated “[the computer] is my working tool in carrying out research. I open many tabs [and] programs [to] work with at the same time, so, my laptop is better than my phone”.

Consistent access to the internet was hypothesized as a potential area where the digital divide might negatively affect people with a disability, people with low incomes, and those living in a LMIC. The significant impact of internet access (under the control of government and business policies) as an environmental conversion factor, was evident, as shown in Table 4. When asked, “Do you have a consistent internet connection?”, location was the only significant result found. Respondents living in Africa were significantly less likely to have a reliable internet connection than those in North America or Europe (*N* = 48, p < 0.001). Income and disability could not be analysed in this regard due to the low number of responses.

**Table 4:** Internet access—survey respondents

	Location n (%)		Income n (%)			Disability n (%)	
	Africa	North America and Europe	Low	Medium	High	Yes	No
*Consistent internet access*							
All the time	7 (21.2)	13 (86.1)	7 (41.2)	1 (25)	12 (57.1)	3 (25)	17 (48.6)
Some of the time	25 (75.8)	2 (13.3)	10 (58.8)	3 (75)	8 (38.1)	9 (75)	17 (48.6)
Never	1 (3)	0 (0)	0 (0)	0 (0)	1 (4.8)	0 (0)	0 (0)
*Method of internet access*							
Fixed cable	1 (3)	7 (46.6)	0 (0)	0 (0)	7 (33.3)	1 (8.3)	7 (20)
Mobile data	30 (90.9)	13 (86.7)	15 (88.2)	3 (75)	20 (95.2)	10 (83.3)	32 (91.4)
Wi-Fi	18 (54.5)	15 (100)	12 (70.5)	3 (75)	17 (80.9)	8 (66.6)	25 (71.4)
*Places to access the internet*							
Home only	6 (18.1)	2 (13.3)	4 (23.5)	0 (0)	1 (4.7)	3 (25)	5 (14.3)
Work only	2 (6.1)	0 (0)	1 (5.8)	0 (0)	0 (0)	2 (16.6)	0 (0)
Home and work	24 (72.7)	13 (86.6)	11 (64.7)	4 (100)	20 (95.2)	7 (58.3)	29 (82.8)
Public place only	2 (6.1)	2 (13.3)	2 (11.7)	4 (100)	0 (0)	10 (83.3)	2 (5.7)
*Can use internet as much as wanted (yes response)*	13 (39.4)	15 (100)	8 (47.1)	2 (50)	16 (76.2)	5 (41.7)	22 (62.9)
*Can use social media as much as wanted (yes response)*	17 (54.8)	15 (100)	9 (56.3)	3 (75)	17 (85)	7 (58.3)	7 (21.2)

When the 48 survey respondents with a known location answered, “Do you use the internet as much as you want?”, all 15/15 (100%) of the HIC respondents said "Yes”, while 13/33 (39%) LMIC respondents said "Yes”. Living in Africa had a significant negative relationship with using the internet as much as wanted compared to North Americans and Europeans, χ^2^(1) = 8.98, p < 0.003, N = 48. Income and disability groups were too small for statistical analysis by location.

Many PIRL members struggled with poor connections for online meetings. Meeting time was used to wait for people to reconnect when connections dropped. Team members commented that they had to remember to pre-charge their devices, because power cuts occurred randomly. Several PIRL researchers had limited ability to contribute to writing in shared documents like a complex spreadsheet or a large document, such as this paper, due to working only on a smaller phone screen (Nganji et al. 2021). Despite individual difficulties, over 300 PIRL meetings and numerous training opportunities were held, three annual institutes conducted, and hundreds of documents created during the life of the PIRL project.

Personal income is an input resource which impacts capability sets. Almost all survey comments related limited income and high costs to an inability to convert ICT resources, like smartphones apps, into desired research capabilities. The survey results about ICT costs were supported by interviewees. For example, interviewee P14 said “connecting online, it does exclude those who can’t afford some of these communication tools”. P03 described assistive technology as “definitely expensive and 9 times out of 10 you have to pay out of pocket”.

The survey results showed that a key environmental conversion factor that impacts individual input resources was the location of participants (Table 3). When access to ICT devices was explored, geographic location was the only significant factor seen in the data available, with 10/33 (30%) from Africa owning 2 or 3 computers, and 9/15 (60%) in North America owning 2 or more computers. All 15/15 (100%) North American and European respondents and 19/33 (58%) respondents from Africa said, “Yes” they could use a computer as much as they wanted. All those who responded “No” when asked if they could use a computer as much as they wanted were living in Africa (14/33, 42%). The Chi-squared results showed a significant association between living in Africa and limits on using a computer (χ2(1), = 8.98, p < 0.003). The interviews corroborated this finding, with comments showing African researchers relied on smartphones for research work and collaboration because their computers, when they had them, often had limited functionality.

### Taking Action: Striving to Increase the Capability Sets of Researchers

The second theme coalesced around the desired and actual actions taken to improve inclusion and increase capability sets, drawing from the PIRL subprojects. Having an explicit focus on taking action to reduce the digital divide effects on team projects, including being able to openly share opinions and suggestions about equity, came up repeatedly in the data and documentation.

Initial discussions at the beginning of the project centred on key researcher personal conversion factors relevant to ICT use including income, gender, education level, and impairments. Social conversion factors considered included ICT training, ICT support availability, access to stipends to cover ICT costs, political strife (resulting in internet and power grid shutdowns), and social policies. Environmental conversion factors included the geographic location of researchers and infrastructure services (e.g., electricity, phone lines, internet broadband, Wi-Fi). Devices and apps - important resource inputs—generated core ICT Plan items; implementing the PIRL ICT plan was a key initial action to achieve desired outcomes such as peer-reviewed papers, conference presentations, increased researcher functionings, and non-academic products.

The ICT Plan was iteratively revised throughout the project. At its core were six guiding principles established early on: access, affordability, value, user experience, availability, and institutional support. In-depth discussions using these principles informed decisions about tools and applications to use, and recognized that for some team members, phones would be their primary device.

The fundamental principle of access aimed to ensure that team members with impairments and limited infrastructure could obtain essential resources and documents. Specialized accessibility apps (e.g. TalkBack) improved inclusion for those with vision loss. Given budget constraints for software licenses, affordability and free apps were key access considerations. We aimed for an optimal user experience for all team members, which led to extensive discussions due to differing personal experiences. Institutional support was considered, as some organizations provided software licenses and application support, while other researchers were excluded because of licensing limitations.

We came to understand that developing and using the ICT Plan was a tool to foster conversation and to increase the capability sets of the researchers, a process which took place at the team level. Using the ICT Plan mitigated some digital divide factors. Similar to Kleine’s recommendations (2011, 2019) to attend to tensions between individual and group capabilities, we found that *intentionally* improving capability sets within the group appeared to have the potential to improve research capabilities not only for individual members, but for the whole team. With training, PIRL researchers in both LMIC and HIC were able to write documents using accessibility standards (e.g., alt text, high contrast, accessible fonts).

The complexity of the ICT Plan to support learning was reflected in survey responses, interviews, and project documents. It was apparent that having access to ongoing training and support could expand capability sets. Some researchers reported that they struggled even when support was available, and there were significant variations in how team members felt supported by the ICT Plan.

This theme also highlights how PIRL members engaged in learning and training processes, (a personal conversion factor), through communicating and collaborating with others. In addition, there were positive comments about access to the PIRL Project technical support person in Cameroon as this kind of support was not available at jobsites. For example, Interviewee P15 described how the PIRL support staff organized workshops, and they valued having someone “always available” when problems came up.

The findings highlight the importance of linking administrative procedures to ICT and digital divide issues. For example, reimbursement of costs to participate in PIRL activities was addressed early on, and core team members who had to pay out of pocket were given the option of having a portion of their costs reimbursed monthly. Others required occasional support for internet access costs. This strategy, which allowed researchers to be able to predict reimbursement, appeared to have a positive impact on engagement in project activities such as webinars, learning institutes, and team meetings, while also adding a layer of administrative challenge. There were many differences within institutions for financial management that created additional administrative challenges, and which were often difficult to solve.

PIRL participants, from both LMIC and HIC, expressed concerns about the cost of using technology to collaborate internationally. Several LMIC survey respondents expressed frustration at having “no internet (even though I have paid for data)”. Another LMIC respondent had 4 internet provider SIM cards and wrote, “despite all of these I cannot guarantee access to [the] internet” reflecting the poor quality of cell service in some locations. One third of the survey comments about access to the internet at work showed that individuals were using their own phone data because their workplace internet service was insufficient.

## Reflecting on the Findings: Maximizing Inclusion in International Research

Here we reflect on the themes from the findings. The first theme described what was learned by examining when, how, and why ICTs were used by researchers in international projects. The second theme summarized the complex ways the team planned and took action to mitigate digital divide barriers and to increase capability sets for all researchers. By focusing on the agency and freedom of researchers to choose research functionings from their capability sets, these PIRL findings extend the CA concept about well-being as freedom to choose opportunities (Sen 2004, 1999). Our results identified that differing access to input resources and differing capability sets can lead to different researcher opportunities, as highlighted by Álvarez-Camacho et al (2021) and Robeyns (2017).

### The Digital Divide and Individual Researchers

As demonstrated by our experience, implementing a survey can be a useful tool to identify the needs of team members and resources available for addressing digital divide barriers specific to each research project. Planning a survey can open conversations that can be potentially difficult, for example about money, skills, cultural differences, and institutional requirements. The results of a survey can identify individual and systemic differences and can direct the planning and administration of team functioning. For example, the survey provided opportunities to discuss how to ask sensitive questions. It was possible in part due to the relatively high educational backgrounds of the respondents; higher than the general population in either a HIC (Canada) (Friesen 2022) or a LMIC (Cameroon) (UNESCO Institute of Statistics 2016) and generally they were in higher income brackets (Belghith 2020; StatsCan Government of Canada 2022), reflecting their interest in research. Other teams might do a survey or gather similar information differently.

The findings from the PIRL subprojects indicated that it was feasible to focus on how smartphones and apps have significant, complex impacts on individual researcher work in the context of team functioning. Key research capabilities included finding and reading relevant research, writing, gathering and analyzing data, and communicating with journals, funders, and community members. PIRL results highlighted that smartphone and internet access improved researcher “freedom to participate” in these tasks, particularly for those at the intersections of low income, disability, and location, thus increasing fairness (Masiero 2022).

Mobile smartphone connections have increased globally, particularly in Africa and Asia (GSMA 2022; 2025b), a trend mitigating digital divides for international researchers. The use of smartphones in place of computers is common. A PEW Research report stated one-quarter of Americans living with low-incomes only use smartphones to access the internet (Vogels 2021). Smartphones are accessible and cost-effective for people with disabilities (Aranda-Jan and Boutard 2019) particularly when training is available to use the built-in accessibility features (GSMA 2025a; International Telecommunication Union of the UN (ITU) 2021c). Examples of researchers who might be choosing to use a smartphone as a main device (in both LMIC and HIC) are graduate students and community researchers, although not well reported in the literature (Amaechi 2024).

Our findings provide a reminder that smartphone access alone is insufficient for all desired research functionings. Robeyns (2017) describes how a resource like a bicycle is affected by conversion factors. Translating the bicycle example to smartphones highlights the different capabilities for different people depending on the conversion factors influencing their use of the resource. For example, if social norms prevent women from using a phone or receiving training, if an environmental lack of electricity or internet prevents use, or a person has low vision without necessary apps, capabilities are reduced. The PIRL results showed that having limited money to pay for environmental conversion factors like data, electricity, and internet services influences the degree to which one can transform a smartphone resource to useful ICT research capabilities, similar to studies such as Faith and Hernandez (2024). The importance of enacting ICT plans and strategies to increase financial inputs was evident in the comments in the survey and during online meetings, which strongly supported the stipend strategy for increasing individual researcher ICT capabilities and research functionings.

Recognizing the complex intersections of impairments, disability, and the digital divide is essential, as “disability” is not binary, and ability to participate varies due to many intersecting factors (Cockburn et al. 2024). For example, we found that non-disabled team members often lacked the knowledge or skills to create inclusive processes and to collaborate with people using assistive devices, a prominent way the digital divide shows up in research teams. In addition, the researchers living with vision impairments needed various technologies which they themselves did not always have access to, perhaps not surprising given the ableism in academic settings, in community-based research, and in society broadly (Brown and Leigh 2018; Wolbring and Lillywhite 2021). These kinds of experiences about the intersections of research and ICT are becoming more visible in the academic literature, for example in reports where the focus is on non-disabled researchers (Hughes et al. 2020; Mellifont et al. 2019).

The PIRL Project members supported the aim of improving each member’s contributions based on their own inputs, conversion factors, capability sets, and choices, reflecting that concerns about fairness, equity, and inclusion can be consistently addressed (Masiero 2022). In situations like those described here, researchers can choose options that increase capabilities for others as well as themselves; PIRL researchers often stated that they were especially concerned for those whose voices most needed to be heard.

### Inclusion, Collaboration, and Digital Divide Effects on Research

Inclusion is essential for collaboration in international development teams and projects (Fransman et al. 2021; Fransman and Newman 2019; Palladino et al. 2023). Scholars have critiqued Sen’s Capability Approach (CA) as overly individualistic, yet a closer examination shows that it incorporates "collective capabilities," where individual empowerment aligns with group efforts, including those aimed at inclusion (Ibrahim 2013; Leßmann 2022; Robeyns 2017). Our results support the concept that inclusivity, equity, and collective capabilities begin with the selection and formation of a team with a diversity of identities and backgrounds. Masiero’s (2022) focus on fairness in the way people are treated is similar to the PIRL approach. Attending to considerations of specific strategies, beginning with who is included, and how they continue to feel and be included as team members, may address concerns such as how more advantaged researchers tend to extract disproportionate value from research paper publications and other outputs (Masiero 2022). Similar to the PIRL Project, Leßmann (2022) suggests that groups embody the concept of collective capabilities: they are internally defined, dedicated to collective well-being, and have goals that can only be achieved through collective effort. Technology is a valuable tool to enhance collective capabilities within global research groups through participation in meetings, conferences, webinars, planning, and production of knowledge resources. Our findings resonate with Ibrahim’s (2013) view that individual actions and group processes mutually reinforce each other to expand capabilities, agency, and freedom.

Research about international development, including the effects of the digital divide, often focuses on populations and averages (International Telecommunication Union of the UN (ITU) 2021a), instead of examining the effects on individuals and teams, overlooking how restricted ICT access affects collective action and collaboration. The PIRL Project, through its participatory research approach to examining the digital divide at both individual and group levels, broadened understandings of collective capabilities and inclusivity. PIRL members required ICTs to engage various stakeholders and embraced a diverse range of voices in creating podcasts, webinars, murals, and theater-based activities extending beyond traditional academic outputs (Animbom et al 2024; Sikapa et al 2024; Nganji et al 2022, 2021). Smartphones were clearly a boon for individual researcher capabilities when computers and internet access were limited.

But the PIRL results make clear that the low income and LMIC participants’ use of smartphones instead of computers was a factor in the decreased quality of collaboration for *all* members of the PIRL team for some tasks. PIRL team members who relied on smartphones struggled with tasks like editing large documents, analyzing spreadsheets, and using multiple apps for simultaneous data collation, consistent with the participants in Faith and Hernandez’s study (2024). Sometimes members met in person to share a computer screen for a task. However, this strategy inadvertently excluded other members who could not attend in person due to the location of the meeting or mobility challenges. These are example of resource inequalities and relational inequalities, which Heeks (2022) describes as crucial components of adverse digital incorporation.

Poor internet connections hindered online meetings, leading to fragmented discussions and limited knowledge exchange, affecting all team members and reducing the quality of collective functionings, despite using audio-only meetings. Extra time was needed to save documents for offline editing and then more time was needed to integrate the suggested edits into shared cloud documents. Time zone accommodations could be challenging.

Collective capabilities were also challenged by individual researcher choices and motivations. Despite the ICT Plan, some researchers chose to use specific software that was inaccessible to others or refused to use applications that the team was using, which reduced collaboration. Overcommitted team members in both HIC and LMIC settings struggled to contribute consistently, and volunteers needed to attend first to income earning activities or their studies.

On the other hand, team researchers recognized, discussed and studied the challenges that were structural and beyond individual control. This finding is in contrast to the work of Ganapathy et al (2024) who illustrated that complex, structural barriers were topics often avoided by researchers. Political conflict, limited infrastructure, fluctuating broadband pricing, and unreliable electricity reveal broader systemic issues affecting collaboration (Lee et al. 2021). It is crucial for international teams to discuss these external constraints, as avoidance can prevent the development of mitigation strategies and lead to personal feelings of guilt or embarrassment. Addressing structural barriers, even if partial, was possible in part due to the diversity of PIRL researchers and the efforts made to recognize and address factors that were beyond individual control, as an attempt to address potential injustices (see, for example, Heeks 2022, for further theoretical discussions of justice and addressing adverse digital incorporation). This understanding highlights a challenge within the CA framework: achieving collective goals requires not only individual agency but also recognition of collective responsibilities to address structural issues.

### Lessons Learned for ICT Planning and Inclusivity in International Research Projects

We propose “lessons learned” for improving ICT use and removing barriers to inclusion and collaboration within research teams. We recognize that this kind of work can be difficult and slow, and the experiences and barriers in other contexts may not be the same as the ones our team faced. Each team needs to develop their own process for addressing equity in ICT needs and opportunities.

We encourage teams to intentionally use technologies to build relationships, maintain hope for more equitable futures (Ganapathy et al. 2024) and celebrate project successes. Part of this work is to actively include researchers who face barriers (Álvarez-Camacho et al. 2021; Ganapathy et al. 2024), to have challenging conversations about disability, financial resources, and ways that researchers can support each other for more equitable access to research resources (e.g. academic databases, institutionally funded software, knowledge of use of accessibility software).

Teams can attend to nuanced cultural and social differences, ensuring that there is a diversity of researchers on the team. Having more perspectives increases strength because intersecting factors related to the digital divide can be explicitly addressed during all stages of a project, including initial design (Masiero 2022). Ensuring that LMIC researchers, women, and researchers with disabilities have key roles can help to prevent key issues from being missed. Attending to nuances can identify barriers, create changes to reduce the barriers, and build trust and respect among members. Recognizing how challenging it can be to have open discussions about the diversity of cultural and socio-economic factors of team members is necessary for honouring the high levels of education and commitment to research and inclusivity made by colleagues in LMICs, which often requires additional learning by those based in HIC (Africa Evidence Network 2021; Álvarez-Camacho et al. 2021). There are many barriers that LMIC researchers must overcome to participate in global teams, which are often not familiar or addressed by HIC colleagues (Africa Evidence Network 2021; Álvarez-Camacho et al. 2021).

Collaboratively and intentionally developing a detailed ICT project plan can open spaces for consideration of individual privileges and challenges, and address misconceptions about ICT access, knowledge, and uses. Explicitly encouraging team members with greater advantages to use their privileges can expand the capability sets of all teammates. ICT Plans can specifically incorporate support for the equitable inclusion of researchers with disabilities. By setting project standards for improving document and website accessibility, the onus for inclusion is placed on those without impairments to practice more inclusive attitudes and actions. One way to help a team to understand inequities is by creating and implementing an ICT survey, which allows for a shared experience. It can help team members understand the disparities across a large team and identify ways to expand collective capabilities.

Explicitly discussing systemic ableism in research, including issues surrounding disability, impairments, and inclusion, and how they intersect with other identities such as gender, income, social class, and geographic location, can improve outcomes. Recognizing that researchers might feel the need to hide disabilities and fear repercussions for being disabled, and that disability costs can be hard to predict, measure, or share requires ongoing and collective engagement to address.

## Limitations

There are some limitations to this study. While efforts were made to include a diverse group, most team members were based in Canada and Cameroon. Additionally, participants were drawn from a single network, and researchers from other locations or teams might have different experiences and perspectives. The small survey sample size (n = 60) limited the depth of quantitative analyses and restricted the breadth of associations and factors we were able to investigate (such as predictors and impact of multiple variables on the outcomes). However, with approximately 100 community members, the 60 completed surveys reflect strong engagement in the PIRL Project. Additionally, requiring all responses in English, regardless of respondents’ first language, may have influenced results; offering surveys in multiple languages could have provided different insights. Understanding the benefits of inclusion of diverse researchers in international projects could be enhanced by increasing the focus on more complex evaluation of outcomes as discussed by Apgar and colleagues (2023), Massett and colleagues (2022), and others (Kaufman et al. 2022; Vogel and Barnett 2023).

This study took place during the COVID pandemic, increasing the need for virtual interaction and reliance on technology, and severely limiting opportunities for face-to-face communication. While not all activities were done virtually, interviews and most meetings were done online, and in-person options might have provided different results, especially for those who experienced difficulties in participating. PIRL members were cognizant of the challenges for those without many technology options or experience, especially in the early days of the project and the pandemic.

## Conclusions

This study illuminated the impacts of the digital divide on international development research team members and offered practical recommendations to improve inclusion. It demonstrated that the use of ICTs for information gathering and communication, both individually and collectively, is fundamental to such projects. Addressing digital divide barriers such as unreliable internet, limited access to functional devices, and inadequate support for assistive technologies can enhance inclusion and expand opportunities for all researchers. The proposed ICT planning strategies and mitigation measures can foster equitable collaboration, reducing disparities caused by geography, income, and disability. Assumptions that are embedded in structures can be changed when team members can talk openly about financial difficulty or excess, and about how to address associated emotional issues, such as impatience, fear, embarrassment, or guilt. Teams can acknowledge that international development research is relational and requires emotional labour by all team members.

The Capability Approach provided a valuable framework for understanding and articulating how improving opportunities and capabilities among diverse researchers can enrich international development efforts. Furthermore, these findings emphasized that inclusive ICT planning not only benefits individual researchers but also strengthens collective research outputs. Researchers and community members have responsibilities for considering how partnership work contributes to improving the world in which we live.

## Data Availability

Data is available through DRYAD. https://doi.org/10.5061/dryad.0cfxpnw8b.
